# Bacterial inclusion bodies as potential synthetic devices for pathogen recognition and a therapeutic substance release

**DOI:** 10.1186/1475-2859-12-16

**Published:** 2013-02-07

**Authors:** Klaudia Talafová, Eva Hrabárová, Dušan Chorvát, Jozef Nahálka

**Affiliations:** 1 Institute of Chemistry, Centre for Glycomics, Slovak Academy of Sciences, Dúbravská cesta 9, Bratislava, SK, 84538, Slovak Republic; 2 Institute of Chemistry, Centre of excellence for white-green biotechnology, Slovak Academy of Sciences, Trieda Andreja Hlinku 2, Nitra, SK, 94976, Slovak Republic; 3 Biophonic Department, International Laser Centre, Ilkovičova 3, Mlynská dolina, Bratislava, SK, 81219, Slovak Republic

**Keywords:** Nanopills, Pathogen targeting, Drug release

## Abstract

**Background:**

Adhesins of pathogens recognise the glycans on the host cell and mediate adherence. They are also crucial for determining the tissue preferences of pathogens. Currently, glyco-nanomaterials provide potential tool for antimicrobial therapy. We demonstrate that properly glyco-tailored inclusion bodies can specifically bind pathogen adhesins and release therapeutic substances.

**Results:**

In this paper, we describe the preparation of tailored inclusion bodies *via* the conjugation of indicator protein aggregated to form inclusion bodies with soluble proteins. Whereas the indicator protein represents a remedy, the soluble proteins play a role in pathogen recognition. For conjugation, glutaraldehyde was used as linker. The treatment of conjugates with polar lysine, which was used to inactivate the residual glutaraldehyde, inhibited unwanted hydrophobic interactions between inclusion bodies. The tailored inclusion bodies specifically interacted with the SabA adhesin from *Helicobacter pylori* aggregated to form inclusion bodies that were bound to the sialic acids decorating the surface of human erythrocytes. We also tested the release of indicator proteins from the inclusion bodies using sortase A and Ssp DNAB intein self-cleaving modules, respectively. Sortase A released proteins in a relatively short period of time, whereas the intein cleavage took several weeks.

**Conclusions:**

The tailored inclusion bodies are promising “nanopills” for biomedical applications. They are able to specifically target the pathogen, while a self-cleaving module releases a soluble remedy. Various self-cleaving modules can be enabled to achieve the diverse pace of remedy release.

## Background

The glycan chains that decorate cell surfaces mediate various normal and pathological processes. They are also responsible for host-pathogen interactions, as they are used by various viruses, bacteria and parasites to promote the pathogenesis. Many pathogens express adhesins on their surface, which are proteins capable of binding to specific glycans. Adhesins play a very important role because the recognition of host-cell oligosaccharides and their adherence is the crucial first step in the colonisation and/or invasion of a pathogen. Moreover, adhesins also determine the tissue tropism of the corresponding pathogen [1,2]. Adhesins have been well-studied in the pathogenesis of *Helicobacter pylori.* This worldwide bacterium causes chronic gastritis, which may result in a peptic ulcer or even in gastritic cancer and mucosa-associated lymphoid tissue lymphoma. Of at least six lectin-like adhesins expressed by *H. pylori*, the most important for colonisation are BabA (blood group-binding adhesin) and SabA (sialic acid-binding adhesin) [3-5]. SabA was selected as a model adhesin for this study.

Recently, the idea has been proposed that the glycoengineering of cell surfaces or the development of specific glyco-nanomaterials will provide new tools for the therapeutic targeting of pathogenic or cancer cells [6,7]. Bacterial inclusion bodies (IBs) have a great potential to serve as nanoparticles for these purposes. IBs are insoluble proteinaceous aggregates commonly observed in bacterial cells during the over-expression of recombinant genes. They have usually been considered to be waste byproducts formed by unfolded or misfolded and thus biologically inactive polypeptides [8]. The few first studies reporting the enzymatic activity of IBs have been ignored [9,10]; however, the Villaverde group (2005) convincingly quantified the biological activity of IBs and proposed an application in bioprocesses [11]. It is already well-known that the formation and disintegration of IBs in the cell is fully reversible and a fraction of IBs is functional. More evidence is appearing, indicating that IBs should not be removed from the bioengineering process. On the contrary, it might be even desirable to target protein production in order to form IBs to be used as biomaterials for industry and biomedicine [8]. Several papers have been published regarding the potential application of IBs in biocatalysis as naturally immobilised enzymes with high stability and good process properties [12-14]. At the same time, their biological origin, mechanical stability and regulatable size make IBs suitable nanomaterials for biomedicine [8].

With regards to biomedicine, the potential application of IBs in tissue engineering and regenerative medicine has been well-studied in the past few years. As it was proven in bottom-up approaches to topographical engineering, IBs decorating the surfaces favour mammalian cell attachment and are capable of stimulating mammalian cell proliferation [15,16]. Moreover, more progressive opinions present IBs as potential “nanopills” for the delivery of therapeutic proteins *via* their extra- or intra-cellular release [17]. Various proteins, including chaperones, enzymes and growth factors, aggregated to form IBs were able to restore relevant missing cell functions without any sign of cytotoxicity [17,18]. Liovic *et al.* (2012) [19] demonstrated the delivery of a polymeric cytoskeletal protein to epithelial cells in the form of soluble IBs; these IBs also did not appear to be cytotoxic. In general, IBs are naturally well-internalised by mammalian cells and it is not unusual for them to reach the nucleus [17]. It is also very encouraging that orally administered IBs did not cause any difficulties in mice models, even when administered in high doses [18].

In the presented work, we have studied the concept of IBs as “nanopills” directly targeting the pathogen surface. First, we demonstrated the basic principle regarding how the tailored IBs are able to specifically recognise the adhesins of pathogens attached to the tissues. Second, we tested the release of indicator proteins representing therapeutic peptides from IBs. We compared the release of soluble proteins from the IBs by protease or intein self-cleavage under conditions of neutral and acidic pH values, respectively. In addition to these results, we achieved the reduction of hydrophobic interactions between the IB particles by the treatment with a polar amino acid.

## Results

### Preparation and testing of tailored IBs

The first step in our work was the preparation of IBs-conjugates representing “nanopills” composed of green fluorescent protein (GFP)-containing IBs (gfpIBs) and soluble proteins using glutaraldehyde as linker. GFP represents a remedy whereas sialylated soluble protein is expected to specifically recognise our model adhesin IBs mimicking a pathogen. Non-sialylated proteins serve as a control to prove the specificity of interactions dependent on sialic acids.

**Figure 1:**
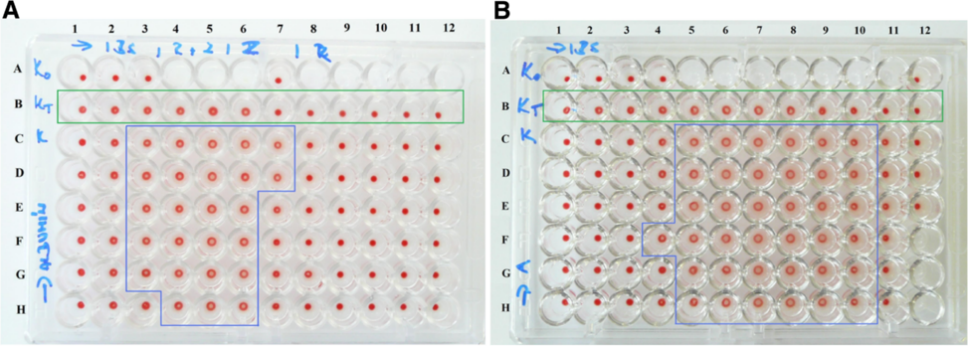
**The reduction of hydrophobic interactions between the conjugated GFP-containing IBs (gfpIBs) and the SabA adhesin aggregated to IBs (sabIBs). A** - gfpIBs conjugated with albumin using nonpolar glycine for glutaraldehyde inactivation**. B** - gfpIBs conjugated with albumin using polar lysine for glutaraldehyde inactivation. Conjugates gfpIBs-albumin (1.25 mg of gfpIBs in 400 μl of PBS) were 1.5-fold diluted from line H to D in 10 μl of PBS. The suspension of sabIBs (isolated from 10 mg of lyophilized transformed *E. coli* in 1000 μl of PBS, diluted in ratio 1:16) was 1.5- (**A**) or 1.2-fold (**B**) serial diluted from column 1 to 12 in 15 μl of PBS. Finally, the wells were titrated with 50 μl of the RBC suspension. Line A - negative control (RBC in PBS), Line B (green frame) - interaction of sabIBs with nonconjugated gfpIBs and RBC, Line C - control interaction of sabIBs with RBC. Blue frame defines the zone of positive hemagglutination.

For the optimisation of the conjugation reaction, the gfpIBs were conjugated with the sialoprotein fetuin and the non-sialylated protein albumin as a control. The prepared conjugates were tested with the hemagglutination-inhibition test. We used SabA lectin from the bacterium *Helicobacter pylori* in the form of IBs (sabIBs) that is responsible for the *in vitro* agglutination of erythrocytes. Because this interaction is dependent on the sialic acid (Sia)-terminated oligosaccharides, it can be modified by sialylated proteins [20], e.g., gfpIBs-fetuin conjugates. We evaluated and compared the extent of the zone of positive hemagglutination of the control and conjugate-testing reactions. However, our results also showed the partial inhibition of hemagglutination by the gfpIB-albumin conjugates at higher concentrations, i.e., the restriction of the zone of positive hemagglutination (Figure 1A). A comparison of the wells containing the sabIBs with the non-conjugated gfpIBs and control wells containing only the sabIBs revealed that the gfpIBs themselves (non-conjugated) inhibited the red-blood-cell (RBC) agglutination. This inhibition is most likely caused by the hydrophobic interactions between the sabIBs and the non-conjugated/conjugated gfpIBs. Consequently, the ability of the sabIBs to bind RBC decreased, which eliminated the prozone effect and increased the interval of negative agglutination (see Figure 1A, compare line B and C). This problem has been solved by the modification of the method used for gfpIB conjugate preparation. The substitution of non-polar glycine by polar lysine in the inactivation of residual glutaraldehyde successfully reduced the hydrophobic adhesion of the conjugated gfpIBs to the sabIBs, to each other and to plastic surfaces. This reduction was reflected by the lack of significant variability in the extent of the positive hemagglutination zone (Figure 1B).

**Figure 2:**
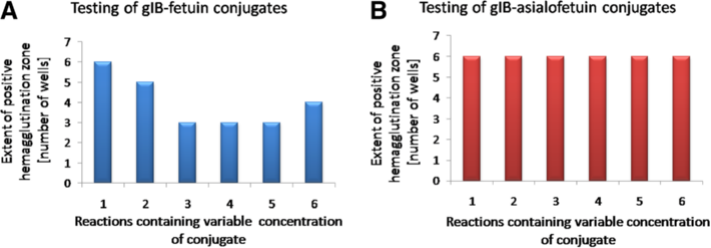
**The testing of the prepared gfpIB-fetuin (A) and gfpIB-asialofetuin (B) conjugates by the evaluation of their impact on the extent of the positive hemagglutination zone.** Hemagglutination-inhibition assay was designed as for optimization (Figure 1). 1 **–** control (sabIBs with RBC, no conjugate), 2–6 – interaction of sabIBs with conjugates and RBC; concentration of gfpIB-fetuin and gfpIB-asialofetuin conjugates is 1.5-fold increasing from 2 to 6.

Once the optimisation was complete, we prepared the gfpIBs conjugated with fetuin and with the non-sialylated representative asialofetuin for further experiments. Testing with the hemagglutination-inhibition assay confirmed the accuracy of the prepared conjugates. The gfpIB-fetuin conjugates inhibited the agglutination of RBC, which was observed as the shortening of the zone of positive hemagglutination compared with control wells. This inhibition was marked, especially at higher concentrations of the conjugate. However, at the highest tested concentration, a modest extension was observed (Figure 2A). In contrast, hemagglutination was not significantly affected by the gfpIB-asialofetuin conjugates, as the positive hemagglutination zone did not vary significantly compared with control wells (Figure 2B).

### *In vitro* visualisation of the specific recognition and binding of tailored IBs to adherent pathogens

**Figure 3:**
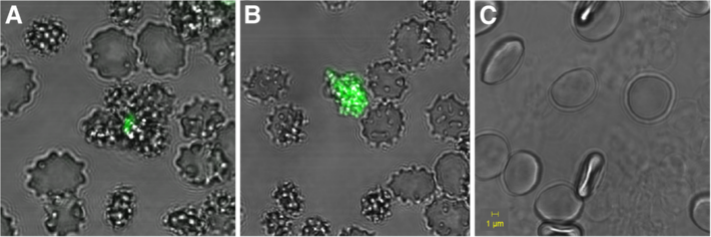
**Images of the *in vitro *model of the specific interactions of the tailored IBs with adherent pathogens. A**) Aggregates of **gfpIBs-fetuin** (green fluorescent particle) are surrounded by erythrocytes carrying SabA adhesin in form of IBs (sabIBs) on their surface – inside of the agglutinates. **B**) Aggregates of **gfpIBs-asialofetuin** (green fluorescent particle) are just randomly attached to erythrocytes with bound sabIBs. **C**) Control represented by erythrocytes in PBS; For better visualization, the micrographs show clusters of gfpIBs particles.

Our prepared conjugates representing “nanopills” suppose to be capable to specifically target an adherent pathogen cell in host organism. To visualise the recognition and binding of these conjugates to pathogen adhesins, we microscopically tested an *in vitro* model. We used the Sia-binding adhesin SabA from *Helicobacter pylori* aggregated to form IBs (sabIBs) which expose on the surface multiple active binding centres. For this reason they are considered as models of pathogen cells. Human stomach tissue is represented by RBC that provide Sia-terminated oligosaccharides on their surface for sabIB binding. Our tailored IB conjugates represent potential particles for the specific targeting of pathogen cells and the delivery of therapeutic proteins. On the basis of results obtained by our testing, we assigned the appropriate concentration of the sabIBs and conjugates. Because our conjugates contain a green fluorescent indicator protein, this interaction was detected using fluorescent confocal microscopy. As seen in the images (Figure 3), the erythrocytes are apparently clustered around the aggregates of gfpIB-fetuin because the sabIBs on their surface also simultaneously interact with the Sias of fetuin in the tailored IBs (Figure 3A). In the case of gfpIB-asialofetuin, the erythrocytes with bound sabIBs are randomly located near aggregated conjugates without any evidence of being clustered inside the aggregates. Asialofetuin does not contain any Sias; thus, there is no multiple-specific interaction observed between erythrocytes and conjugates (Figure 3B).

### A remedy released from inclusion bodies

**Figure 4:**
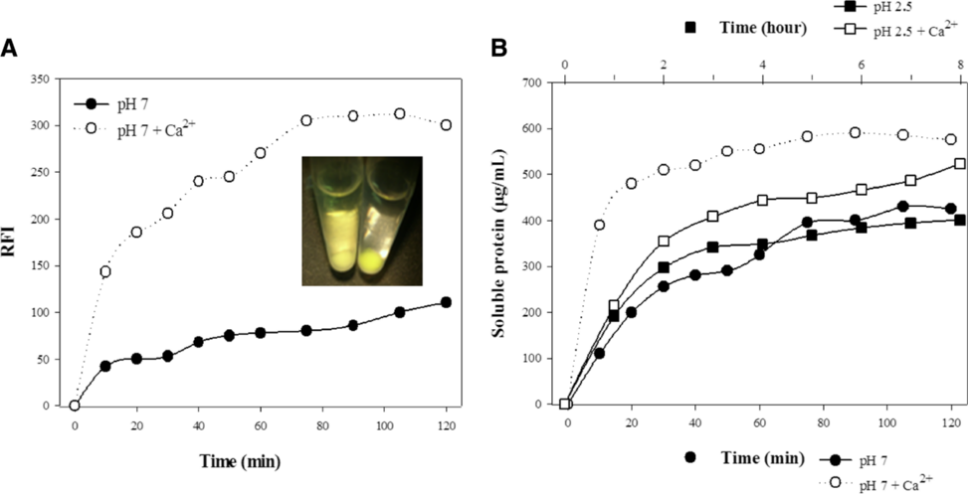
**GFP released from the inclusion bodies composed of the CBDclos-SrtA-GFP fusion protein. A**) Fluorescence of soluble protein released from IBs under neutral pH. The inset shows colors of supernatant and sediment before (on the right) and after (on the left) release. RFI - relative fluorescence intensity **B**) Concentration of soluble protein released from IBs under acidic and neutral pH. Total concentration of the CBDclos-SrtA-GFP fusion protein was 5.42 mg/ml.

**Figure 5:**
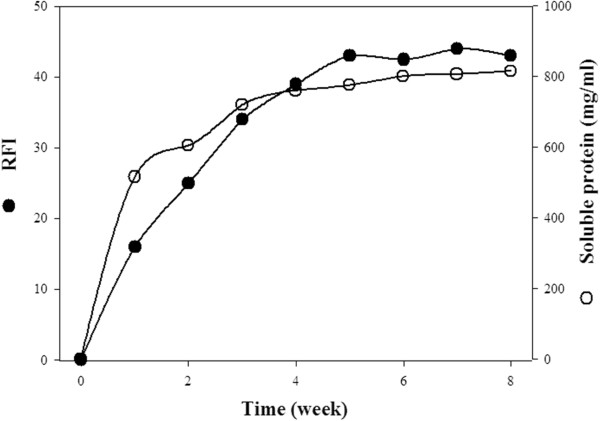
**GFP released from the inclusion bodies composed of the CBDclos-intein-GFP fusion protein.** Total concentration of the CBDclos-intein-GFP fusion protein was 6.86 mg/ml.

Firstly, our goal was to demonstrate the release kinetics governed by the two model self-cleaving modules under two model pH conditions. To explore more precisely possibilities of the release regulation, we tested the release potential of “remedy-carrying” compounds of unconjugated IBs. Thus, we eliminated any factors (e.g. soluble parts of conjugates) that might bring additional complications by influencing the release kinetics and their primary comparison. For this purpose, we tested two unconjugated constructs: CBDclos-SrtA-GFP and CBDclos-intein-GFP, despite the fact that conjugated CBDclos-intein-GFP IBs – gfpIBs were used in previous sections. The cellulose-binding domain from *Clostridium cellulovorans* (CBDclos) serves as a 20 kDa “pull-down domain” that pulls down expressed proteins, changing them from a soluble to an insoluble form while maintaining the activity of the fused protein [12]. The GFP represents the potential remedy which should be released from the particles. It was chosen to achieve better visualisation and measurement of the process (Figure 4A, inset). *Staphylococcus aureus* sortase A (SrtA) and Ssp DNAB intein were inserted between the CBDclos and GFP and serve as cleaving modules; both are capable of cleaving the protein chain at its *C*-terminus and release the otherwise soluble GFP. SrtA is protease specific for the LPXTG amino acid sequence and needs Ca^2+^ ions for optimal activity [21]. The Ssp DNAB intein from *Synechocystis* sp. has temperature- and pH-dependent self-cleavage activity at its C-terminus [22]. These fusion proteins were designed for fast, medium, slow and very slow activities. Figure 4 shows the fast (pH 7, activated by Ca^2+^) and medium (pH 7) releases, which can be counted in minutes. Interestingly, the time course of the relative fluorescence intensity was “slower” than the measured protein concentration in the supernatant, indicating that GFP folding is completed after its release into the solution. According to the potential application for the targeting of bacterial cells in the stomach, the activity was tested at an acidic pH (2.5). In this case, SrtA showed moderate activity but was still active. The GFP release was quite slow and, for this reason, can be counted in hours. The GFP was not capable of fluorescence at pH 2.5; therefore, the released protein was monitored only by its protein concentration in the supernatant (Figure 4B). The CBDclos-intein-GFP protein aggregates showed very weak cleavage efficiency; the time course was measured in weeks (Figure 5). Both types of physiologically aggregated constructs were dissolved in 1% SDS and the protein concentration was measured using a modified biuret reagent for the Lowry procedure (TP0200 and B 3934, Sigma, Germany). According to the soluble protein concentrations shown in the graphs (Figures 4 and 5), the CBDclos-SrtA-GFP protein aggregates released a maximum of 10.7% whole aggregated protein in 90 minutes, while the CBDclos-intein-GFP protein aggregates released 11.8% whole aggregated protein in 8 weeks.

## Discussion

In this work, we present proof of principle of a novel direction of protein drug delivery in medicine. Our experimental results show that correctly tailored IBs could be cheap protein cassettes for protein drug delivery that are able to specifically attack pathogen adhesins or receptors of different tissue. Additionally, the tailored IBs are able to carry and release a protein in “programmable” time courses.

Our tailored IBs were prepared *via* a conjugation reaction using glutaraldehyde. The IBs containing the indicator protein GFP (CBDclos-intein-GFP) were linked to a sialylated and non-sialylated protein, respectively. To test this reaction, the resulting conjugates were tested using our method based on the hemagglutination-inhibition assay [20]. The Sia-dependent hemagglutination is expected to be inhibited by the conjugates containing fetuin but unaffected by the asialofetuin conjugates. However, because the hemagglutination was evoked by the SabA adhesin in the form of IBs, the hydrophobic interactions between the sabIBs and gfpIBs, apparently also between sabIB and gfpIB conjugates, misrepresented the results of our test. Generally, bacterial IBs, as opposed to soluble globular proteins, represent physiological aggregates composed mainly of recombinant proteins in the form of an unfinished tertiary structure; therefore, more hydrophobic acids are exposed on the water-protein interface, thereby allowing themselves IBs formation and clustering IBs to large clusters by hydrophobic interactions. In other words, the ratio of the burial of hydrophobic residues from water balances the aggregate stability and enzyme activity in IBs. Ideally in our fusion constructs, the CBDclos module provides the maximal hydrophobic interaction among fusion protein molecules and forms maximally stable aggregates, and the cleaving module, together with GFP, form the maximal globule stage and provide the maximal activity. However, the hydrophobic interactions between the sabIBs and gfpIBs were not desired; therefore, we decided to change the amino acid used for glutaraldehyde inactivation in the preparation of the gfpIB conjugates. Glutaraldehyde reacts very well with various amino acids [23]; thus, they are used as deactivators of un-reacted glutaraldehyde [24,25]. For our conjugation reaction, glycine was the first choice for the glutaraldehyde inactivation because it is a small neutral amino acid without any significant effects on the intramolecular hydrophobic interactions [26]. After observing the unwanted hydrophobic interactions between the sabIBs and conjugates, we applied an excess of lysine to eliminate the un-reacted glutaraldehyde. Lysine is largely polar [26], and treatment with it “polarised” the surface of the IBs and successfully eliminated the hydrophobic interactions between the sabIBs and gfpIB conjugates as well as between the gfpIBs and plastic surfaces. After applying this approach, our test confirmed the specificity of the Sia-dependent interactions between erythrocytes, sabIBs and conjugates. As expected on the basis of on our previously published results [20,27], the fetuin-containing conjugates modified the level of hemagglutination, whereas the asialofetuin-containing conjugates maintained the hemagglutination level similar to control wells.

The specificity of the interactions was also confirmed by our *in vitro* model. We selected the pathogenic bacterium *Helicobacter pylori* and its SabA adhesin as a model for our study. The SabA adhesin has been well-studied in the pathogenesis of *H. pylori*. Because it recognises Sias, it binds the sialylated antigens on the inflamed gastric epithelium and on RBC in gastric mucosal blood vessels [3,4]. SabA aggregated to form IBs is substituting pathogen cells in our model. Under a fluorescent confocal microscope, it was confirmed that only the tailored IBs containing fetuin strongly interacted with the pathogen cells (represented by sabIBs) bound to the Sias on the erythrocyte surface. The conjugates containing asialofetuin did not show the same interactions under the same conditions. The conjugates used for this experiment were compressed into a pellet due to centrifugation and resuspension of the pellet with a pipette resulted in the formation of smaller clusters. These clusters were used for the microscope reaction due to better visibility. However, the basic concept is the application of the IBs as “nanopills” for biomedicine. Several works have been already published, demonstrating that IBs are mechanically stable enough to tolerate the ultrasonication needed to obtain IBs with a median spherical diameter of 200–500 nm [28,29]. Thus, conjugates could be very easily broken into nanoparticles *via* sonication. Our results provide convincing evidence that tailored IBs are potentially able to specifically target adherent pathogen cells on human tissues. Erythrocytes in our *in vitro* model underwent some morphological changes compared to control erythrocytes in neutral phosphate buffer. RBC are very susceptible to the environment changes, and the transformation of their shape might be caused by various aspects [30]. We suggest that morphological changes are a consequence of binding of sabIBs to the Sias on the cell surface. However, this has no significant impact on our results, as this shape modification was observed in a model of the interaction of RBC-sabIBs with gfpIB-fetuin as well as with gfpIB-asialofetuin.

The other part of this study focused on demonstrating the drug release from gfpIBs. The therapeutic protein is represented by GFP, the release of which was tested under two pH conditions. Neutral pH (pH 7.0) was chosen as the primary condition under which also specific pathogen recognition *in vitro* was demonstrated. However, the pH values in the human body vary widely. Because a model adhesin for our recognition study was SabA from *H. pylori*, a bacterium colonising stomach, we decided to test also protein release under acidic pH conditions (pH 2.5). The GFP remains fluorescently active when aggregated into the IBs, as was demonstrated by fusion to the VP1 capsid protein of the foot-and-mouth disease virus [11]. We used the CBDclos system that initiates the physiological aggregation of GFP and also maintains protein activity. As a cleaving module, we used the *S. aureus* sortase A (SrtA) and Ssp DNAB intein from *Synechocystis* sp. SrtA has already proven to be an efficient tag for the purification of recombinant proteins when fused to their N-termini [21,31] but, until now, was not applied to the protein release from IBs. Our results show that the SrtA protease is capable of effectively releasing the protein from IBs at a neutral pH in a relatively short period of time. At an acidic pH, such as in the stomach, the progress of GFP release was markedly slower compared to that observed at neutral pH. Nevertheless, the very slow (several weeks lasting) release of the proteins was observed in the aggregates containing intein as the cleaving module. The Ssp DNAB intein was tested as a part of the potential cleavable self-aggregating tag, indicating the aggregation of the target proteins to form IBs and the subsequent intein-cleavage and release of soluble protein. However, the cleavage efficiency of this intein was marked as insufficient; thus, it was not tested further [32]. Although its cleavage activity is not suitable for protein purification, this intein might be beneficial for biomedical purposes, as its sustained, long-lasting drug release might be desirable in some cases, such as in the treatment of chronic diseases [33]. In contrast, the treatment of some infections requires an instantaneous high dose of a drug [34], indicating the sequential drug release over several minutes to hours. And this can be achieved by the protease cleavage activity. Nowadays, various mechanisms of controlled drug release are described and their application depends on the way of administration as well as the type of infection. However, novel concept of nanocarriers enabling the drug targeting to specific cells is considered as very beneficial [35].

## Conclusions

The results presented here, combined with the knowledge of pathogen adhesins, can provide an effective tool for direct pathogen targeting with therapeutic substances. IBs are currently considered to be promising nanoparticles for biomedical purposes. The tailored IBs used in this study were composed of indicator proteins aggregated to form IBs that were conjugated with soluble proteins responsible for pathogen recognition. The indicator proteins forming the IBs represented a remedy. The conjugates during preparation were treated with a polar amino acid to avoid the hydrophobic interactions between the IBs in subsequent experiments. The results confirmed our hypothesis that the IBs are able to specifically recognise and bind to the surface of adherent pathogens. The use of two self-cleaving modules, protease and intein, also demonstrated that the indicator proteins forming the IBs can be efficiently released from the IBs and thus converted from an insoluble into a soluble form. The protease provided fast protein release, taking from several minutes to hours depending on the conditions, whereas the intein-cleaving module enabled a sustained protein release over several weeks. The model proposed here represents a promising advance in biomedicine, introducing IBs as potential “nanopills” able to specifically attach to pathogen adhesins and subsequently release a remedy. Each of the two tested self-cleaving modules provides a different pace of remedy release that might be chosen depending on the particular infection.

## Material and methods

### Cloning, expression and isolation of SabA adhesin aggregated to form IBs (sabIBs)

The method described by Nahalka et al. (2009) [27] was used for the production of SabA lectin in the form of active IBs. IBs were isolated from *Escherichia coli* BL21(DE3) transformed by plasmid vector pET-34b(+). The insert carried by vector was truncated gene HP0662 isolated from genomic DNA of *Helicobacter pylori* ATCC700824D. This gene was inserted in such a way that N-terminus of the resulted protein was fused with the cellulose-binding domain of *C. cellulovorans* (CBDclos). The described fusion initiated physiological aggregation of the protein into the active IBs. Transformed *E. coli* was cultivated in LB medium (10 g/l trypton, 5 g/l yeast extract, 10 g/l NaCl) with kanamycine (30 μg/ml). After cultivation, the cells were lyophilized. IBs were isolated from 10 mg of lyophilized cells using 500 μl of non-ionic lytic detergent (Sigma, B7435-500ML). The lysate was subsequently centrifuged (13000 g, 10 min., 4°C) and washed three times in 750 μl Tris–HCl (50 mM, pH 7.5). Finally, the pellet was suspended in 1 ml of PBS (0.8% NaCl, 0.02% KCl, 0.115% Na_2_HPO_4_x7H_2_O, 0.02% KH_2_PO_4_, pH 7.2).

### Cloning, expression and isolation of *Staphylococcus aureus* sortase A plus GFP and Ssp DNAB intein plus GFP aggregated to form IBs (gfpIBs)

The GFP gene was amplified by PCR from TurboGFP plasmid purchased from EVRΩGEN. *Staphylococcus aureus* sortase A (SrtA) and Ssp DNAB intein (intein) genes were artificially synthesized by GenScript Corporation. The AGGCCT restriction places for StuI enzyme have been attached to the C-terminus of synthesized genes. The synthetic genes were inserted into pET-34b(+) plasmid, the constructs were linearized by StuI enzyme, and subsequently, GFP sequence has been inserted to the vectors by LIC method. As the result, constructs for the expression of CBDclos-SrtA-GFP and CBDclos-intein-GFP fusion proteins were obtained. CBDclos-SrtA-GFP fusion protein contained KKLPETGR linker sequence inserted between SrtA and GFP. The cultivation of transformed *Escherichia coli* BL21(DE3) and the isolation of IBs were performed as mentioned above, but in the case of CBDclos-SrtA-GFP fusion protein, 15 mM EDTA was added to lytic detergent and washing buffer.

### Preparation of fetuin-glutaraldehyde-IBs conjugate

A glycoprotein fetuin (*M*_w_ = 48.4 kDa) (Sigma, F2379-1 G), asialofetuin (Sigma, A4781-250MG) or albumin (Sigma, A4503-10 G) (both reference samples) – 25 mg/250 μl – were conjugated *via* glutaraldehyde (GAL; 0.25%) with IBs. IBs were, prior to reaction, prepared as above-mentioned in the suspension concentration of 1.25 mg/375 μl (PBS; pH 7.2). IBs-GAL conjugate was prepared as follows: a cooled stock GAL solution was gradually added in 10 μl aliquots under stirring (by pipette) in 10-min intervals during the 1^st^ h to achieve a final GAL concentration of 0.25% and final volume 435 μl. The whole reaction time was 2 h/+4°C. Fetuin was under intensive stirring quickly dissolved in the reaction system. The reaction proceeded 1 h/+4°C. The same procedure was performed applying asialofetuin or albumin. An amino acid – l-glycine or l-lysine (to achieve 100 mM in final volume 485 μl) was added at the end of the conjugation reaction (blocking the unreacted -CHO groups). A molar concentration ratio of amino acid/GAL was ~ 3.8. The system was then left to stand at +4°C until next day. It was finally washed and centrifuged three times with PBS and prepared for the reaction with erythrocytes and sabIBs.

### Testing of tailored IBs

Prepared conjugates gfpIBs-fetuin/gfpIBs-asialofetuin or gfpIBs-albumin were resuspended in 400 μl of PBS. First, the conjugates gfpIBs-fetuin and gfpIBs-albumin were tested just to optimize conjugation reaction. After optimization, the conjugates gfpIBs-fetuin and gfpIBs-asialofetuin were prepared and tested. The method used for testing is based on hemagglutination-inhibition test and was described by Talafová and Nahálka (2012) [20]. The conjugate suspension was 1.5-fold diluted in 10 μl of PBS in microtiter plate from line H to D. The suspension of sabIBs was diluted in ratio 1:16, the resulting suspension was 1.5- or 1.2-fold serial diluted in PBS and added to conjugates in volume of 15 μl. Finally, the mixture was titrated with 50 μl of the RBC suspension (50 μl of fresh human blood in 5 ml of PBS). RBC were voluntarily donated by one of the authors (KT). The RBC suspension in PBS was considered as a negative control. The other controls contained RBC suspension with (i) sabIBs and nonconjugated gfpIBs or (ii) sabIBs alone. The microtiter plate was incubated for 1 hour at room temperature and subsequently 2 hours at 4°C. After incubation, electronic record was used to compare tested samples with control wells.

### *In vitro* visualisation of specific recognition and binding of tailored IBs to adherent pathogen

RBC suspension (500 μl) was mixed with 150 μl of sabIBs suspension diluted in ratio 1:32 and additional 100 μl of PBS. After short gentle shaking, RBC were allowed to sediment. After supernatant removal, the sediment was washed twice with PBS, suspended in 500 μl of PBS and mixed with 66.7 μl of the suspension of conjugate IBs-fetuin or IBs-asialofetuin, and additional 183.3 μl of PBS by gentle shaking. RBC were allowed to sediment, and after supernatant removal, they were washed twice with PBS. Finally, the sediment was suspended in 500 μl of PBS and observed by fluorescent confocal microscopy. RBC were voluntarily donated by one of the authors (KT).

### Soluble protein release from unconjugated gfpIBs

IBs composed from CBDclos-SrtA-GFP fusion protein were resuspended in the 50 mM phosphate buffer (5.42 mg/ml) alternatively containing 2 mM Ca^2+^ (pH 7.0) or 5 mM Ca^2+^ (pH 2.5), and IBs composed from CBDclos-intein-GFP fusion protein were resuspended in the 50 mM Tris buffer (6.86 mg/ml, pH 7.0). The stock suspension was divided into equal parts which were kept at +4°C before withdrawal to determine time-course of the soluble protein release. The samples were withdrawn and centrifuged in time-intervals depicted in Figures 4 and 5. Each point was evaluated in triplicate. RFI (excitation wavelength 482 nm; emission wavelength 502 nm) and A_280_ values were obtained using Fluorimeter (BioTek FLx 800TM Multi-Detection Microplate Reader, Germany) and Spectrophotometer (Infinite M200 TECAN, Switzerland). Fluorescence was not measured at the acidic pH, because IBs became colorless.

A colorimetric method utilizing *Total Protein Kit* composed of *Biuret Reagent* and *Folin and Ciocalteu’s Phenol Reagent* was used to determine soluble protein concentration (mg/ml) in the supernatant (TP0200 and B 3934, Sigma, Germany). Absorbance at 750 nm was obtained spectrophotometrically, and the protein concentration was plotted against the time. A total protein concentration of the IBs at the time t_0_ was evaluated after dissolving in 1% SDS. However, it is worth of noticing that the CBDclos-intein-GFP-containing IBs were less easily soluble in 1% SDS – prepared in 0.85% NaCl – than the CBDclos-SrtA-GFP-containing IBs.

### Consent

KT (one of the authors) provides consent for publication of this report and any accompanying images.

## Competing interests

The authors declare that they have no competing interests.

## Authors’ contributions

KT performed the hemagglutination-inhibition assay, *in vitro* model of pathogen targeting and wrote the paper. EH isolated sabIBs, gfpIBs, performed conjugation with fetuin or other proteins, and realised soluble protein release experiments. DCh performed confocal microscopy. JN constructed the expression plasmids, designed the concept of the manuscript, and edited the manuscript. All authors read and approved the final manuscript.
